# Two cases of life‐threatening arrhythmia induced by risperidone: evaluation of risperidone and 9‐hydroxy‐risperidone concentrations

**DOI:** 10.1002/ams2.277

**Published:** 2017-04-24

**Authors:** Asami Ito, Tomoyuki Enokiya, Eiji Kawamoto, Yoshiaki Iwashita, Taichi Takeda, Kenji Ikemura, Masahiro Okuda, Hiroshi Imai

**Affiliations:** ^1^ Emergency and Critical Care Center Mie University Hospital Tsu Japan; ^2^ Department of Pharmacy Mie University Hospital Tsu Japan

**Keywords:** Complete AV block, overdose, QT prolongation, risperidone

## Abstract

**Cases:**

Case 1: A 20‐year‐old woman suffering a suspected overdose was transported to the hospital. She presented bradycardia with wide QRS waves and QT prolongation, followed by cardiac arrest. Extracorporeal cardiopulmonary resuscitation was implemented, improving circulation. Risperidone and 9OH‐RIS levels were 9.6 ng/mL and 127.6 ng/mL, respectively. Case 2: A 54‐year‐old woman was hospitalized for femoral fracture and underwent surgery. Her electrocardiogram showed bradycardia and complete AV block. Risperidone and 9OH‐RIS levels were 3.2 ng/mL and 61.4 ng/mL, respectively.

**Outcome:**

In both cases, only serum concentration of 90H‐RIS were elevated.

**Conclusion:**

Arrhythmia related to risperidone has proven rare but sometimes fatal. Serum concentrations of risperidone and the metabolite 9‐hydroxy‐risperidone (9OH‐RIS) during these events are seldom documented. As 9OH‐RIS shows pharmacological activity equivalent to risperidone, it may induce life‐threatening arrhythmia (regardless of the intake dosage). It is critical to evaluate the serum concentration of 9OH‐RIS in suspected risperidone toxicity.

## Introduction

Risperidone is a second‐generation antipsychotic commonly used in patients with schizophrenia. It is a serotonin 5‐HT_2A_ receptor antagonist, which relieves both positive and negative symptoms. However, over‐prescription and inappropriate use have recently become problematic issues.

In 2005, the US Food and Drug Administration published a safety statement warning against off‐label use of antipsychotic drugs for dementia, stating their association with a 1.6 to 1.7‐fold increase in mortality in elderly patients.^1^ Nevertheless, second‐generation antipsychotics are still widely used. According to a study in Australia, antipsychotic overdoses are increasing, with second‐generation antipsychotic overdoses doubling during the past 26 years.^2^

Despite this, few cases have documented the serum concentrations of risperidone and its active metabolite 9‐hydroxy‐risperidone (9OH‐RIS) during fatal arrhythmia induced by the drug. The present study describes two patients with severe arrhythmia induced by risperidone. In both cases, only serum concentrations of 9OH‐RIS were elevated. These findings suggest that the serum concentration of 9OH‐RIS is a significant indicator of risperidone toxicity.

## Case 1

A 20‐year‐old woman found unconscious at home was transported to the emergency room (ER). She had a history of depression, and was taking the medications risperidone, flunitrazepam, and aspirin. It is estimated that she took 93 tablets of risperidone (122 mg), 25 tablets of flunitrazepam (50 mg), and 160 tablets of aspirin (52.8 g). Following arrival at the ER, she was immediately intubated due to respiratory depression. Her blood pressure was 80/45 mmHg, heart rate was 120 b.p.m., and Glasgow Coma Scale score was E4VTM4. Electrocardiogram (ECG) showed sinus tachycardia, with a corrected QT interval (QTc) of 455 ms. Results of blood gas analysis were: pH 7.28; pCO_2_, 43.0 mmHg, HCO_3_, 20.3 mmol/L; and K, 5.5 mmol/L. After activated charcoal was given, her blood pressure dropped, and the ECG showed bradycardia with wide QRS waves, and eventually pulseless electrical activity. Chest compressions were started, followed by extracorporeal cardiopulmonary resuscitation. The patient regained spontaneous circulation, and was admitted to the intensive care unit. Therapeutic hypothermia was implemented, and she was taken off extracorporeal cardiopulmonary resuscitation the following day. Serum concentrations of risperidone, 9OH‐RIS, and aspirin were 9.6 ng/mL, 127.6 ng/mL, and 756 μg/mL, respectively. Serum concentration was analyzed using the liquid chromatography tandem mass spectrometry technique. The patient was extubated on day 12, and subsequently transferred to another hospital.

## Case 2

A 54‐year‐old woman's car plunged off a cliff and she was transported to the ER. She had no major injuries except for a right femoral fracture. She had no medical history and no medications. She underwent surgery for her leg on day 24 of admission, and was then transferred to the orthopedics ward. On day 29, her ECG monitor alarm sounded for irregular activity and the emergency physician was called. The patient was alert and had normal vital signs. A recorded ECG revealed a complete AV block. The patient was prescribed risperidone on day 12 of admission due to symptoms of delirium, and the dose was increased to 3 mg 2 days prior to the event. She was also taking famotidine and magnesium oxide. The patient was transferred to the intensive care unit for observation, and risperidone was discontinued. Her blood tests revealed normal risperidone serum concentration (3.2 ng/mL) and elevated 9OH‐RIS serum concentration (61.4 ng/mL), thereby confirming risperidone toxicity. Arrhythmia was not documented following discontinuation of risperidone.

## Discussion

Here, we report two cases of severe arrhythmia associated with 9OH‐RIS. There are few published reports of severe arrhythmia stemming from risperidone toxicity in which the serum concentration of risperidone and 9OH‐RIS were documented (Table 1). In the present cases, only 9OH‐RIS levels were high, whereas the level of risperidone fell within the normal range.

**Table 1:** Serum concentrations of risperidone and 9‐hydroxy‐risperidone (9OH‐RIS) in previous reports of arrhythmia or sudden cardiac arrest induced by risperidone

Authors	Year	Age, years / gender	ECG presentation	Dose of risperidone, mg	Time after risperidone intake to blood test, h	Risperidone serum concentration, ng/mL	9OH‐RIS serum concentration, ng/mL
Ito *et al*.	2016	28/Female	QT prolongation, PEA	122	<6	9.6	127.6
	2016	54/Female	Complete AV block	3	<10	3.2	61.4
Pollak *et al*.^3^	2011	33/Female	QT prolongation	60	5	>100	>1000
Lee *et al*.^4^	1997	21/Female	QT prolongation	100	4	1070	100
Springfield *et al*.^5^	1996	45/Male	Sudden cardiac arrest	403	Unknown	1800 ng/mL	–

ECG, electrocardiogram; PEA, pulseless electrical activity. (–), not assessed.

Risperidone inhibits the potassium ion channel current *I*_Kr_, which is involved in ventricular repolarization and the QT interval. Risperidone also has the ability to inhibit the human ether‐a‐go‐go‐related gene (*hERG*) channel current, a recombinant channel encoded by an *I*_Kr_‐cloned equivalent. Vigneault *et al*.^6^ showed in animal models that 9OH‐RIS also inhibits the *hERG* channel current, prolonging QT intervals. In our first case, we concluded that the high serum 9OH‐RIS concentration led to prolonged QTc and cardiac arrest. Although salicylate toxicity was initially suspected, other possibilities were investigated for the following reasons. First, the severe metabolic acidosis and respiratory alkalosis commonly observed in fatal salicylate toxicity were mild in the present case, and unlikely to cause cardiac arrest. Second, salicylate poisoning often causes sinus tachycardia, but fatal ventricular arrhythmias are rare (electrolyte abnormality being the most common cause). However, the serum potassium level was only slightly elevated. In the second case, risperidone toxicity was diagnosed based on serum concentrations and exclusion of other causes such as cardiac disease and other drug interactions. The patient's slightly hypovolemic state may have caused risperidone toxicity despite a standard dose, however this remains unclear.

Risperidone is metabolized to 9OH‐RIS in the liver by the cytochrome enzymes CYP2D6 and CYP3A4. As the metabolite is pharmacologically similar to risperidone, the clinical effect of the drug is evaluated by the active moiety (the sum of risperidone and 9OH‐RIS). Plasma elimination half‐lives of risperidone and 9OH‐RIS in extensive metabolizers are 2.8 and 20.5 h, respectively. The half‐life for the active moiety is approximately 24 h.^7^ 9‐Hydroxy‐risperidone is mostly dominant in serum, measuring approximately 22 times higher than that of risperidone.^8^ The therapeutic and toxic ranges of the two compounds are not well established, although AGNP Consensus Guidelines recommend combined levels of risperidone and 9‐OH‐RIS at 20–60 ng/mL.^9^

Recent studies show that 9OH‐RIS may have a central role in adverse effects. Suzuki *et al*.^10^ evaluated the association between risperidone metabolism and QTc interval, and found a significant positive correlation between plasma 9OH‐RIS levels and the QTc interval, whereas there was no correlation between plasma risperidone levels and QTc. These findings imply the importance of the serum concentration of 9OH‐RIS.

The technique used to analyze serum concentrations of the two compounds is not readily available in all medical facilities. However, as previously mentioned, 9OH‐RIS elongated the QTc interval in a concentration‐dependent manner. Overdose patients or patients with a high risk of toxicity may benefit from these measurements. Easier accessibility is anticipated, given the wide prescription of the drug.

In both cases, we were unable to attain genotype information, and further investigation is required. However, in conclusion, we propose that the serum concentration of 9OH‐RIS is a clinically relevant indicator for risperidone toxicity. It is instructive to evaluate the serum concentration of 9OH‐RIS when risperidone toxicity is suspected.

## Conflict of Interest

None declared.
